# Subchronic *N*-acetylcysteine Treatment Decreases Brain Kynurenic Acid Levels and Improves Cognitive Performance in Mice

**DOI:** 10.3390/antiox10020147

**Published:** 2021-01-20

**Authors:** Tonali Blanco Ayala, Daniela Ramírez Ortega, Paulina Ovalle Rodríguez, Benjamín Pineda, Gonzalo Pérez de la Cruz, Dinora González Esquivel, Robert Schwarcz, Korrapati V. Sathyasaikumar, Anabel Jiménez Anguiano, Verónica Pérez de la Cruz

**Affiliations:** 1 Graduate Program in Experimental Biology, DCBS, Universidad Autónoma Metropolitana-Iztapalapa, Ciudad de Mexico 09340, Mexico; tonaliblaya@gmail.com; 2 Neurosciences Area, Biology of the Reproduction Department, Universidad Autónoma Metropolitana, Ciudad de México 09340, Mexico; aja@xanum.uam.mx; 3 Neurochemistry and Behavior Laboratory, National Institute of Neurology and Neurosurgery “Manuel Velasco Suárez”, Mexico City 14269, Mexico; drmz_ortega@hotmail.com (D.R.O.); paulina.ovalle.rodriguez@gmail.com (P.O.R.); dinora.gonzalez@innn.edu.mx (D.G.E.); 4 Neuroimmunology Department, National Institute of Neurology and Neurosurgery “Manuel Velasco Suárez”, Mexico City 14269, Mexico; benjamin.pineda@innn.edu.mx; 5 Department of Mathematics, Faculty of Sciences, Universidad Nacional Autónoma de México, UNAM, Mexico City 04510, Mexico; gonzalo.perez@ciencias.unam.mx; 6 Maryland Psychiatric Research Center, Department of Psychiatry, University of Maryland School of Medicine, Baltimore, MD 21228, USA; rschwarcz@som.umaryland.edu (R.S.); Saikumar@som.umaryland.edu (K.V.S.)

**Keywords:** *N*-acetylcysteine, kynurenic acid, kynurenine, learning and memory

## Abstract

The tryptophan (Trp) metabolite kynurenic acid (KYNA) is an α7-nicotinic and *N*-methyl-d-aspartate receptor antagonist. Elevated brain KYNA levels are commonly seen in psychiatric disorders and neurodegenerative diseases and may be related to cognitive impairments. Recently, we showed that *N*-acetylcysteine (NAC) inhibits kynurenine aminotransferase II (KAT II), KYNA’s key biosynthetic enzyme, and reduces KYNA neosynthesis in rats in vivo. In this study, we examined if repeated systemic administration of NAC influences brain KYNA and cognitive performance in mice. Animals received NAC (100 mg/kg, i.p.) daily for 7 days. Redox markers, KYNA levels, and KAT II activity were determined in the brain. We also assessed the effect of repeated NAC treatment on Trp catabolism using brain tissue slices ex vivo. Finally, learning and memory was evaluated with and without an acute challenge with KYNA’s bioprecursor L-kynurenine (Kyn; 100 mg/kg). Subchronic NAC administration protected against an acute pro-oxidant challenge, decreased KYNA levels, and lowered KAT II activity and improved memory both under basal conditions and after acute Kyn treatment. In tissue slices from these mice, KYNA neosynthesis from Trp or Kyn was reduced. Together, our data indicate that prolonged treatment with NAC may enhance memory at least in part by reducing brain KYNA levels.

## 1. Introduction

Kynurenic acid (KYNA) is a neuroactive metabolite of the kynurenine pathway (KP), the major route of degradation of the essential amino acid tryptophan (Trp) [1]. Since KYNA crosses the blood–brain barrier very poorly [2], brain KYNA levels are determined to a large extent by local synthesis from its brain-penetrant bioprecursor L-kynurenine (Kyn). In the mammalian central nervous system (CNS), four different enzymes have been shown to be able to catalyze this transamination [3,4,5], with kynurenine aminotransferase II (KAT II) being considered the principal isoform in both in human and rodents [6,7,8].

At high concentrations, KYNA is a competitive inhibitor of all ionotropic glutamate receptors [9,10,11]. At low micromolar concentrations, and therefore of possible physiological significance, KYNA inhibits the obligatory glycine co-agonist site of the NMDA receptor and, non-competitively, the alpha-7 nicotinic acetylcholine receptor (α7nAChR) [11,12]. Since both NMDA and α7nACh receptors have well-established roles in cognition [13,14,15,16], endogenous KYNA may be involved in cognitive performance [17]. Experimental manipulation of KYNA levels in the adult rodent brain causes a spectrum of cognitive deficits including impairments in prefrontal-mediated cognitive flexibility and hippocampus-mediated contextual learning and memory [18], as well as working memory and contextual fear memory [18,19,20,21,22]. Moreover, genetic deletion of kynurenine 3-monooxygenase (KMO) in mice, which causes a shift in KP metabolism leading to a several-fold elevation in brain KYNA levels, results in deficits in contextual memory and increased anxiety [23]. In contrast, the reduction in KYNA levels seen in the brain of KAT II knockout mice has pro-cognitive consequences, leading to improved performance in several behavioral paradigms (object exploration and recognition, passive avoidance, and spatial discrimination) as well as increased long-term potentiation assessed electrophysiologically in the hippocampus [24]. Notably, and in conceptual agreement, acute pharmacological reduction of KYNA by selective KAT II inhibitors significantly improves cognitive performance in rats [25,26,27].

Aging, neurodegenerative diseases, and psychiatric disorders have been repeatedly shown to be associated with elevated brain KYNA levels as well as cerebral redox disturbances, evidenced by high levels of reactive oxygen species (ROS), decreased activity of antioxidant enzymes, and reduced glutathione (GSH) content [28,29]. ROS play a significant role in brain KP metabolism [30] since as direct or indirect actions of ROS provide alternative routes of KYNA neosynthesis in non-vertebrate models [31,32]. In 2015, our group demonstrated that this mechanism can also account for the formation of KYNA in the rodent brain in vivo [33]. Thus, we suggested that antioxidants may represent an attractive, new pharmacological approach to reduce KYNA production in the brain.

*N*-acetylcysteine (NAC), a thiol-containing compound and known antioxidant [34], is frequently used as an adjuvant in the treatment of neurodegenerative diseases and psychiatric disorders [34,35,36,37]. The clinical benefits of NAC, which are mostly seen after prolonged treatment, are commonly ascribed to the ability of the drug to bolster the body’s defenses against oxidative stress, infection, toxic assaults, and inflammatory conditions, and to regulate glutamatergic and dopaminergic neurotransmission [38,39,40]. In experimental models of premature aging, NAC administration resulted in improved cognition [41,42], prevented cognitive disturbance in a model of Alzheimer’s disease [43] and ameliorated behavioral and motor deficits in the R6/1 model of Huntington’s disease [44,45].

We recently reported that the beneficial actions of NAC may involve a reduction in KYNA synthesis. Specifically, we showed that NAC inhibits KAT II activity in brain tissue homogenates from both rats and humans, interferes with the de novo formation of KYNA in rat brain tissue slices, acutely reduces KYNA neosynthesis in the rat prefrontal cortex in vivo [46], and competitively inhibits recombinant human KAT II (Ki: 450 µM). The present in vivo study, performed in mice, was designed to examine the functional and translational relevance of this molecular mechanism by assessing the effects of subchronic NAC administration on cerebral KP metabolism and cognitive performance.

## 2. Materials and Methods

### 2.1. Animals

Male FVB/N mice, obtained from the vivarium of the National Institute of Neurology and Neurosurgery (Mexico City, Mexico), were used for this study. The animals (5 per cage) were housed in acrylic cages and provided with a standard commercial rodent diet (Laboratory rodent diet 5001, PMI Feeds Inc., Richmond, IN, USA) and water ad libitum. All mice were housed in the same room under identical environmental conditions, i.e., temperature (25 ± 3 °C), humidity (50 ± 10%), and lighting (12 h light/dark cycles).

All procedures with animals were carried out according to the National Institutes of Health Guide for the Care and Use of Laboratory Animals and the local guidelines on the ethical use of animals from the Health Ministry of Mexico. All efforts were made to minimize animal suffering during the study.

### 2.2. Materials

Pyruvate, pyridoxal-5′-phosphate, Kyn, KYNA, 3-hydroxykynurenine (3-HK), NAC, GSH, oxidized glutathione (GSSG), aminooxyacetic acid, thiobarbituric acid (TBA), and 2′,7′-dichlorodihidrofluoresceine diacetate (DCF-DA) were obtained from Sigma-Aldrich Company (St. Louis, MO, USA). All other chemicals were of the highest commercially available purity. Solutions were prepared using deionized water obtained from a Milli-RQ (Millipore, Burlington, MA, USA) purifier system.

### 2.3. Subchronic NAC Treatment

Ten-week-old mice were administered NAC (100 mg/kg, i.p.) or saline solution daily at 8 a.m. for 7 days (20 animals per group; total 40 mice). The brains of 10 mice per group (10 saline and 10 NAC) were cut into two halves; one half was used for the preparation of tissue slices (ex vivo assays) and the other half for biochemical analyses (KAT II and KMO activity, KYNA and 3-HK levels, GSH, GSSG, lipid peroxidation, and ROS production) as detailed below. Due to experimental issues, two mice from each group could not be used for the ex vivo experiments.

The remaining 20 mice (10 saline and 10 NAC) were used for behavioral analyses (see below).

### 2.4. Tissue Collection

Mice were euthanized by decapitation 6 h after the last i.p. injection. Brains were rapidly removed, and the cortex was dissected out on ice. For experiments using brain tissue slices, 1 × 1 mm blocks were prepared using a razor blade, and the tissues were immediately immersed in freshly oxygenated Krebs–Ringer buffer (118.5 mM NaCl; 4.75 mM KCl; 1.77 mM CaCl_2_; 1.18 mM MgSO_4_; 5 mM glucose; 12.9 mM NaH_2_PO_4_; and 3 mM Na_2_HPO_4_; pH 7.4). All other tissues were rapidly frozen on dry ice and stored at −80 °C until analysis.

### 2.5. Analytical Procedures

#### 2.5.1. GSH and GSSG Levels

For the determination of GSH and GSSG levels, brain tissue from 10 animals per group (saline and NAC) was weighed while frozen and then homogenized (1:10, *w*/*v*) in buffer A (154 mM KCl, 5 mM diethylenetriamine penta-acetic acid, DTPA) and 0.1 M potassium phosphate buffer, pH 6.8. Following addition of cold buffer B (40 mM HCl, 10 mM DTPA, 20 mM ascorbic acid and 10% trichloroacetic acid), the homogenates were centrifuged (14,000× *g*, 20 min). For GSH determination, 5 µL of the supernatant was combined with o-phthaldialdehyde (OPA) to obtain the isoindole. For GSSG determination, 30 µL of the homogenate was mixed with 4 µL of *N*-ethylmaleimide (7.5 mM final concentration) to neutralize GSH. Subsequently, GSSG was reduced to GSH using 6 µL of sodium hydrosulfite (100 mM final concentration). Finally, derivatization with OPA was performed to obtain the isoindole. The isoindole was measured by fluorescence at 370 (excitation) and 420 nm (emission) using a Synergy™ HTX multi-mode microplate reader (Biotek Instruments, Winooski, VT, USA). Results are expressed as nanomoles of GSH or GSSG/g of tissue [47].

#### 2.5.2. Determination of Lipid Peroxidation (LP) and ROS

Brain tissue from 10 animals per group (saline and NAC) was weighed while frozen and then homogenized (1:10, *w*/*v*) in Krebs–Ringer buffer (pH 7.4). Lipid peroxidation (LP) and ROS were assessed simultaneously in the tissue homogenates incubated alone or with FeSO_4_ (5 µM) for 2 h at 37 °C in a water bath. LP was evaluated by the reaction of TBA reactive species (TBA-RS) and malondialdehyde (MDA), one of the final products of polyunsaturated fatty acid peroxidation in the cells. Following incubation, the samples were boiled with 250 µL of TBA (0.375 g TBA and 15 g trichloroacetic acid (TCA) + 2.54 mL HCl in 100 mL) in a water bath for 15 min. Samples were then placed on ice and centrifuged (12,000× *g*, 10 min). MDA was determined as a colorimetric product using Synergy™ HTX multi-mode microplate reader (Biotek Instruments) at a wavelength of 532 nm. Results were expressed as micromoles of MDA per mg protein.

ROS were evaluated through DCF-DA oxidation. After addition of DCF-DA (75 μM final concentration), brain homogenate was incubated for 30 min at 37 °C in darkness. Samples were then centrifuged (9000× *g*, 10 min), and ROS formation was quantified in the supernatant using fluorescence spectrophotometry (Synergy™ HTX multi-mode microplate reader, Biotek Instruments) at an excitation wavelength of 448 nm and an emission wavelength of 532 nm. Results are expressed as a percentage of ROS production vs. controls.

#### 2.5.3. KAT II Activity and KYNA Levels

For determination of KAT II activity, brain tissue from 10 animals per group (saline and NAC) was weighed while frozen and then homogenized (1:10, *w*/*v*) by sonication (Branson Ultrasonics Corp., Danbury, CT, USA) in Krebs buffer (pH 7.4). Tissues were further diluted 1:2 (*v*/*v*) in 5 mM Tris-acetate buffer (pH 8.0) containing pyridoxal-5′-phosphate (50 µM) and 2-mercaptoethanol (10 mM). One hundred µL of the homogenate was then incubated for 2 h at 37 °C with Kyn (final concentration: 100 µM) in Tris-acetate buffer (150 mM, pH 7.4) containing pyruvate (1 mM) and pyridoxal-5′-phosphate (80 µM) in a total volume of 200 µL. Blanks were obtained by the addition of aminooxyacetic acid (1 mM final concentration). The reaction was stopped by the addition of 50 µL of 50% (*w*/*v*) trichloroacetic acid and 1 mL of 0.1 M HCl. After suitable dilution, 50 µL of the solution was applied to a 3 µm C_18_ reverse phase column (ZORBAX Eclipse XDB 5 µm, 4.6 × 150 mm; Agilent, Santa Clara, CA, USA), and KYNA was isocratically eluted using a mobile phase containing 250 mM zinc acetate, 50 mM sodium acetate, and 3% acetonitrile (pH 6.2) at a flow rate of 1 mL/min. In the eluate, KYNA was detected fluorometrically (excitation wavelength: 344 nm; emission wavelength: 398 nm; S200a fluorescence detector; Perkin-Elmer, Waltham, MA, USA). The retention time of KYNA was 7 min.

For the determination of endogenous KYNA levels, brain tissue was thawed and homogenized in deionized water (1:5, *w*/*v*). Twenty-five µL of perchloric acid (6%) was added to 200 µL of the samples. After through mixing, the precipitated proteins were removed by centrifugation (14,600× *g*, 10 min). Fifty µL of the resulting supernatant was subjected to HPLC analysis, and KYNA was assessed as described above.

#### 2.5.4. KMO Activity and 3-HK Levels

Brain tissue from 10 animals per group (saline and NAC) was weighed while frozen and then homogenized (1:10, *w*/*v*) in Krebs buffer (pH 7.4). The homogenate was diluted (1:2, *v*/*v*) in 100 mM Tris-HCl buffer (pH 8.1) containing 10 mM KCl and 1 mM EDTA. One hundred µL of the preparation was incubated for 2 h at 37 °C in a solution containing 1 mM NADPH, 3 mM glucose-6-phosphate, 1 unit/mL glucose-6-phosphate dehydrogenase, 100 µM Kyn, 100 mM Tris-HCl buffer (pH 8.1), 10 mM KCl, and 1 mM EDTA in a total volume of 200 µL. The reaction was stopped by the addition of 50 µL of 6% perchloric acid. Blanks were obtained by boiling the samples for 10 min. After centrifugation (14,600× *g*, 10 min), 100 µL of the supernatant were applied to a 3 µm Adsorbosphere C18 reverse phase column (4.6 × 100 mm; Thermo Fisher Scientific, Waltham, MA, USA), using a mobile phase consisting of 1.5% acetonitrile, 0.9% triethylamine, 0.59% phosphoric acid, 0.27 mM EDTA, and 8.9 mM sodium heptane sulfonic acid and a flow rate of 0.6 mL/min. In the eluate, the reaction product, 3-HK, was detected electrochemically using a LC-4C detector (BAS, West Lafayatte, IN, USA) oxidation potential: +0.5 V). The retention time of 3-HK was ~11 min.

For the determination of endogenous 3-HK levels, brain tissue was thawed and homogenized (1:10, *w*/*v*) in ultrapure water. Twenty-five µL of 6% perchloric acid was added to 200 µL of the samples. After thorough mixing, the precipitated proteins were removed by centrifugation (14,600× *g*, 10 min). One hundred µL of the resulting supernatant was subjected to HPLC analysis, and 3-HK was assessed as described above.

### 2.6. Experiments Using Tissue Slices

Nine cortical slices (3 slices to be incubated under basal conditions, 3 to be exposed to Trp, and 3 to be exposed to Kyn) from each of the 8 animals per group (saline and NAC) were used. Each set of 3 slices was placed in a well of a Falcon 24-well cell culture plate containing 500 µL of Krebs–Ringer buffer (pH 7.4), and the tissues were incubated either under basal conditions or in the presence of Trp or Kyn (both 1 mM) for 2 h at 37 °C. The culture plate was then immediately placed on ice, and 500 µL of the incubation medium was transferred to 1.5 mL Eppendorf tubes. Fifty µL of 6% perchloric acid was added, the samples were centrifuged (14,600× *g*, 10 min), and the resulting supernatant was diluted as needed. Fifty μL of the supernatant was then subjected to HPLC for KYNA and 3-HK analysis (see above). KYNA and 3-HK production from Trp and Kyn was determined after the deduction of basal values.

### 2.7. Protein

Where indicated, protein was determined according to the method of Lowry et al. [48] using bovine serum albumin as a standard.

### 2.8. Behavioral Tests

#### 2.8.1. Buried Food Location Test (BFLT)

An investigator who was unaware of the experimental protocol performed all behavioral experiments. BFLT is an adaptation [49] of the model described by Lehmkuhl et al. for the evaluation of olfactory dysfunction [50]. Since acute Kyn administration models cognitive impairment (see Introduction), four groups of animals were used: (1) subchronic saline, (2) subchronic NAC, (3) subchronic saline + acute Kyn, and (4) subchronic NAC + acute Kyn. Mice in groups 3 and 4 received an i.p. injection of Kyn (100 mg/kg) 60 min before the training session (acquisition) on Day 6. This session consisted of 6 trials (2 min inter-trial interval) and a “0” trial in which the mouse was placed in an acrylic box (1 m^2^), covered with a 3 cm-sawdust layer and highly palatable “fruit loops” (sugary pellets to which they were previously familiarized) buried 1 cm under the sawdust in a fixed quadrant of the box. The location of the pellet was the same in all trials, and the box had spatial cues (black color geometric figures of 10 × 10 cm, which were placed in the middle of each face of the box at a height of 13 cm). Before the training session, the animals were fasted for 24 h, with *ad libitum* access to water. If mice were unable to find the fruit loop within 180 s in the first trial, they were gently guided to it (the fruit loop was placed on the surface). The mice were allowed to eat the fruit loop for 5 s. After each trial, the animals were returned to their home cage, the testing area was cleaned with a 10% ethanol solution, and the sawdust was removed in order to eliminate odoriferous marks. Memory was evaluated 24 h after the acquisition session in a 3 min retention test where the food was removed from the sawdust, and mice were allowed to freely explore the arena for 180 s. The time needed to reach the precise location of the buried food (same location as during acquisition) was recorded, and the exploration time spent in the food location quadrant was measured. All sessions were video-recorded, allowing offline analysis with a tracking software (ImageJ, Bethesda, MD, USA). The results are expressed as the time to reach the target and the time spent searching for the food.

#### 2.8.2. Motor Activity

Locomotor activity was evaluated in all animals immediately after the memory test using an Opto-Varimex 4 system (Columbia, OH, USA). Briefly, the motor/kinetic activity was recorded during 5 min after the habituation period, and the cage was cleaned with alcohol between each test. Results are expressed as total distance walked (cm) and ambulatory time (s) recorded in 5 min, as previously reported [49]. After behavioral tests, brain was dissected out for KYNA determination (see above).

### 2.9. Statistical Analyses

All data are expressed as the mean ± SEM. Two-way ANOVA was performed with pairwise comparisons using Tukey’s adjusted *p*-values, and a logarithmic transformation was used. When comparing only two groups (NAC vs. saline), a Mann–Whitney test was performed. Spearman’s rank correlation was used for analyzing the association between two numerical variables. Values of *p* < 0.05 were considered significant.

## 3. Results

### 3.1. Subchronic NAC Administration Reduces Lipid Peroxidation and ROS Production Caused by the Pro-Oxidant FeSO_4_ but Does Not Increase GSH Levels in the Brain

**Figure 1:**
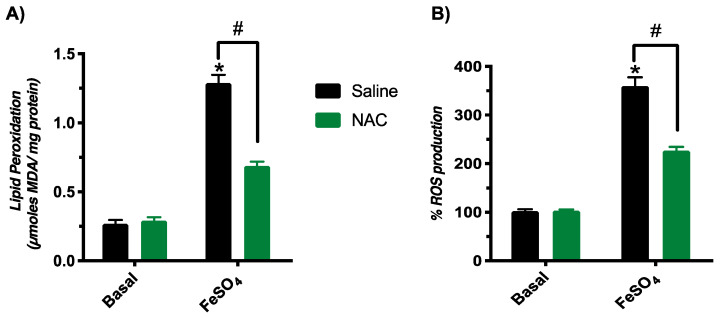
Effect of subchronic NAC administration on brain lipid peroxidation and ROS production induced by the pro-oxidant FeSO_4_ in vitro. Brain tissues were obtained from mice treated i.p. with saline or NAC (100 mg/kg/day). See Materials and Methods for experimental details. (**A**) Basal status of lipid peroxidation and effect of FeSO_4_ (5 µM). (**B**) ROS formation, expressed as a percentage of control values, under basal conditions and after exposure to FeSO_4_. Data are the mean ± SEM (*n* = 10 for each of the 4 groups). Two-way ANOVA was performed for lipid peroxidation and ROS with pairwise comparisons using Tukey’s adjusted *p*-values. * *p* < 0.05 vs. basal saline and ^#^ *p* < 0.05 vs. saline + FeSO_4_.

We first assessed the effect of subchronic NAC administration on the brain’s redox status, based on the treatment schedule reported by Raza et al. [51] (daily i.p. injection of 100 mg/kg NAC for 7 days). As illustrated in Figure 1, basal lipid peroxidation and ROS production were similar between animals treated with saline and NAC. However, acute exposure to the pro-oxidant FeSO_4_ caused significantly less lipid peroxidation and reduced ROS formation (by 46 ± 3% and 37 ± 2%, respectively) in tissue homogenates from mice pre-treated with NAC compared to saline-treated controls. These results, which are in line with the data reported by Raza et al. [51], confirm the protective effects of NAC pre-treatment against an acute oxidative insult.

In the same groups of animals, we observed no differences in the brain levels of either GSH or GSSG between NAC-treated mice and saline-treated control animals (Table 1).

### 3.2. Subchronic NAC Administration Decreases KYNA Levels, Reduces KAT II Activity and Raises 3-HK Levels in the Brain

**Figure 2:**
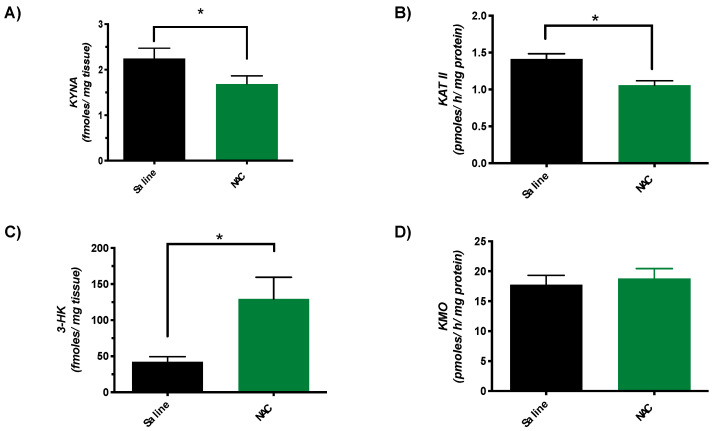
KYNA levels (**A**), KAT II activity (**B**), 3-HK levels (**C**), and KMO activity (**D**) in the brain (cortex) of mice after subchronic administration of NAC (100 mg/kg/day) or saline. See Materials and Methods for experimental details. Data are the mean ± SEM of 10 animals per group, * *p* < 0.05 vs. saline (pairwise comparisons, Mann–Whitney test).

Since acute administration of NAC reduces KYNA neosynthesis [46], we next determined KYNA levels and KAT II activity in the brain of animals that were treated subchronically with NAC. As illustrated in Figure 2, cortical KYNA levels were ~25% lower than in saline-treated controls (1.7 ± 0.2 vs. 2.3 ± 0.2 fmoles/mg tissue) (Figure 2A). KAT II activity was similarly affected, showing a reduction by ~21% in mice that had received NAC (1.1 ± 0.1 vs. 1.4 ± 0.1 pmoles/h/mg protein) (Figure 2B). Parallel measurements of 3-HK levels and KMO activity revealed a substantial increase in brain 3-HK (129.3 ± 30.1 vs. 42.4 ± 7.1 fmoles/mg tissue) after subchronic NAC treatment but no change in KMO activity (17.8 ± 1.6 vs. 18.8 ± 1.6 pmoles/h/mg protein).

### 3.3. Subchronic NAC Treatment Reduces KYNA Neosynthesis in Brain Tissue Slices Ex Vivo

**Figure 3:**
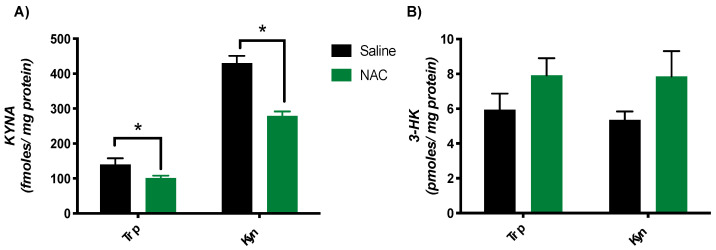
Effect of subchronic NAC treatment on the neosynthesis of KYNA (**A**) and 3-HK (**B**) in brain tissue slices. Tissues were obtained from mice treated i.p. with NAC (100 mg/kg/day) or saline and were then incubated with Trp or Kyn (each 1 mM) in vitro, as described in Materials and Methods. Data are the mean ± SEM (*n* = 8 per group); * *p* < 0.05 vs. saline (pairwise comparisons, Mann–Whitney test).

The next experiment was designed to examine the neosynthesis of KYNA and 3-HK from Trp or Kyn (1 mM each) using cortical tissue slices obtained from mice treated with NAC or saline for 7 days. Tissues from animals treated with NAC had a reduced capacity to produce KYNA from both Trp (NAC: 101.4 ± 6.7 fmoles/mg protein; saline: 140.3 ± 17.5 fmoles/mg protein) and Kyn (NAC: 278.9 ± 12.8 fmoles/mg protein; saline: 430.5 ± 20.7 fmoles/mg protein) (Figure 3A). Tested in the same tissue samples, 3-HK production from both precursors showed a trend to increase as a result of the NAC treatment, but the differences from saline-treated controls did not attain statistical significance (* *p* > 0.05; Mann–Whitney test) (Figure 3B).

### 3.4. Subchronic NAC Administration Improves Memory Performance

**Figure 4:**
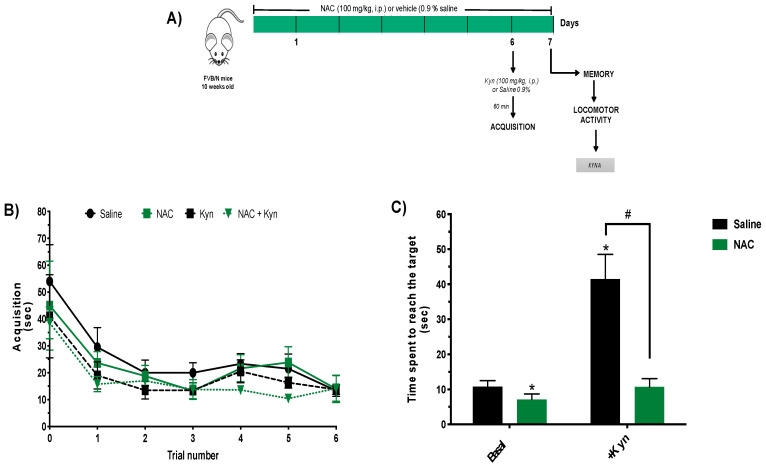
Subchronic NAC administration improves memory in the buried food location test. (**A**) Schematic illustration of the experimental protocol memory procedure. Memory and locomotor activity were examined after the subchronic administration of NAC (100 mg/kg/day) or saline. Two groups of animals received an additional acute i.p. injection of Kyn (100 mg/kg) or saline before acquisition of spatial learning on Day 6. See Materials and Methods for experimental details. (**B**) Acquisition (training) on Day 6. (**C**) Memory performance on Day 7. Data are the mean ± SEM (*n* = 5 per group). Two-way ANOVA was performed for memory with pairwise comparisons using Tukey’s adjusted *p*-values: * *p* < 0.05 vs. saline and ^#^ *p* < 0.05 vs. Kyn.

Finally, we investigated whether the reduction in brain KYNA levels caused by subchronic administration of NAC affects performance in the buried food location test (Figure 4A). Mice receiving NAC or saline did not differ with regard to acquisition of spatial learning on Day 6 (Figure 4B). When memory was evaluated 24 h later, NAC-treated animals reached the target location significantly faster than saline-treated controls (7.1 ± 1.6 and 10.8 ± 1.7 s, respectively) (Figure 4C).

To further examine the effect of subchronic NAC administration on cognitive performance, separate groups of mice received an injection of Kyn (100 mg/kg, i.p.) 60 min prior to the training session on Day 6 to acutely raise cerebral KYNA levels, which are known to disrupted learning and memory process [19]. Mice injected with Kyn (i.e., subchronic saline + Kyn and subchronic NAC + Kyn) showed no difference in learning (acquisition), i.e., the animals reduced the time spent to reach the target location in a similar fashion (Figure 4B). However, memory performance was severely impaired in subchronic saline + Kyn animals; the time spent to reach the target location was almost three times higher (41.5 ± 7.1 s) than in controls (10.8 ± 1.7 s). Notably, however, Kyn did not impair memory in mice that had been pretreated with NAC (41.5 ± 7.1 vs. 10.7 ± 2.3 s, respectively) (Figure 4C).

**Figure 5:**
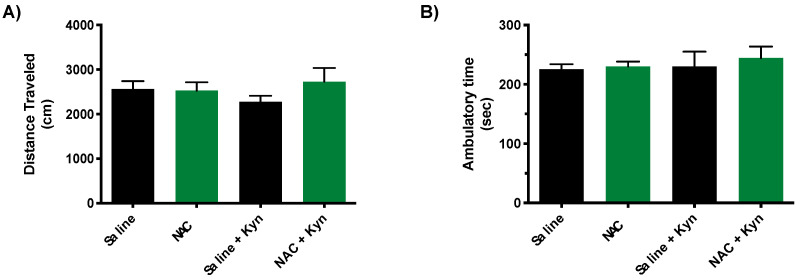
Locomotor activity in mice following the memory test on Day 7 (cf. Figure 4A). Distance traveled and ambulatory time are showed in (**A**) and (**B**), respectively. See Materials and Methods for experimental details. Data are the mean ± SEM (*n* = 5 per group). No significant group differences were observed (*p* > 0.05, Mann–Whitney test).

Locomotor activity was tested promptly after the memory test on Day 7. No changes were observed between groups in the distance traveled and ambulatory time (Figure 5). 

**Figure 6:**
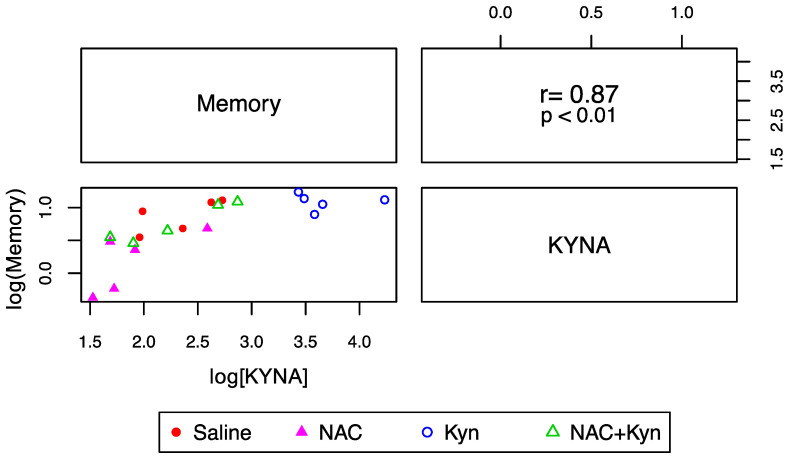
Correlation between KYNA levels and memory performance. The lower triangular matrix contains the scatterplot for KYNA (fmoles/mg tissue) and the time (seconds) the animal spends to find the target (memory). The upper triangular matrix contains the Spearman’s rank correlation coefficient (r) and its associated *p*-value (*p*) (*n* = 5 per group).

Finally, we analyzed whether the effect of NAC on KYNA levels correlated with cognitive performance. As illustrated in Figure 6, there was a positive association between reduced brain KYNA levels and improved memory performance (expressed as the time spent to find the target), suggesting that subchronic NAC administration improves memory performance and prevents the cognitive impairment induced by an acute Kyn administration at least in part by decreasing brain KYNA levels.

## 4. Discussion

The present set of experiments, performed in mice, was designed to explore the effect of repeated systemic NAC injections on brain KYNA levels and neosynthesis and a possible relationship to the drug’s beneficial effect on memory [52,53]. We first confirmed that daily application of 100 mg/kg NAC for 7 days provided protection against an acute pro-oxidant challenge even though the redox status of the brain remained unchanged under basal conditions. Since the favorable clinical effects of NAC are generally attributed to increased formation of the antioxidant GSH [54], we next determined the brain content of GSH (and its oxidation product GSSG) after subchronic NAC administration. The fact that the tissue levels of both GSH and GSSG remained unchanged prompted us to explore a separate mechanism with biological significance, namely a possible effect of the treatment on the brain levels of the neuromodulator KYNA. This concept was based on our recent in vitro and in vivo studies showing that NAC—but not GSH—acutely reduces KYNA formation by inhibiting its key biosynthetic enzyme, KAT II [46].

After noting that subchronic NAC administration indeed reduces KYNA levels as well as KAT II activity in the brain, we demonstrated that this treatment also interfered with KYNA formation in an ex vivo paradigm, namely in freshly dissected cortical tissue slices that were acutely incubated with either Trp or Kyn, the two most pivotal KP constituents [1]. The fact that the effect of Trp and Kyn in slices from mice pretreated with NAC was shifted away from KYNA neosynthesis and toward increased 3-HK production (presumably due to increased availability of Kyn for enzymatic conversion by KMO) is in line with the effects of acute NAC injections on KYNA production [46] and suggests that functional amounts of NAC were retained in brain tissues excised from mice after the subchronic administration of the drug.

Causative links between reduced brain KYNA levels and enhanced cognitive performance have been demonstrated repeatedly in a large number of preclinical studies and are now well established (see Introduction). Our present finding of improved memory in mice receiving daily injections of NAC for 7 days is in excellent agreement with this notion. Further conceptual support was provided by testing memory function in NAC-pretreated mice that received a single systemic injection of Kyn, which rapidly raises KYNA levels in the brain [19], 60 min before the acquisition phase on Day 6. Compared to animals pre-treated with saline, where acute Kyn treatment resulted in substantial cognitive impairment, mice that had received NAC were protected against the deterioration in memory function. Notably, the functional benefit of NAC, which may involve KYNA-induced effects on dopaminergic, glutamatergic, and GABAergic neurotransmission [17], was unrelated to the learning pattern during the training session and to impaired locomotor activity.

Taken together, the present data show that the cognitive improvement caused by pharmacological and genetic manipulation of KYNA levels can also be achieved by repeated systemic administration of NAC, a readily accessible drug with an excellent safety profile [35,40]. Of note, the pro-cognitive effect of NAC shown here and elsewhere may not only be related to the drug’s ability to inhibit KAT II and reduce brain KYNA levels but could additionally involve redox mechanisms [55]. Interestingly, NAC may also influence KYNA formation by interfering with an alternative, oxidative route of KYNA neosynthesis [31,33]. These mechanisms need to be studied in detail and may become especially relevant in diseases where redox disturbances contribute to cognitive impairments [56,57,58].

## 5. Conclusions

In conclusion, the present study provides direct evidence that NAC-induced cognitive improvement is at least partly caused by the drug’s ability to reduce KYNA levels in the brain. Our work suggests that this mechanism may play a critical role in the beneficial effects of NAC in clinical settings, including brain disorders associated with oxidative stress, neuroinflammation, and other neurochemical abnormalities.

## Figures and Tables

**Table 1:** Brain levels of GSH and GSSG after subchronic NAC (100 mg/kg per day) administration. Data are the mean ± SEM (*n* = 10 per group). No significant group differences were observed (Mann–Whitney test).

	GSH (nmoles/g Tissue)	GSSG (nmoles/g Tissue)
**Saline**	3910.7 ± 26.7	444.7 ± 12.8
**NAC**	3947.5 ± 31.7	388.8 ± 14.9

## Data Availability

Data used to support the findings of this study are available with the corresponding author upon request.
